# COVID-19 from the perspective of otorhinolaryngology: An analysis of bibliometrics

**DOI:** 10.3389/fpubh.2022.1002686

**Published:** 2022-09-23

**Authors:** Ji Wang, Sai Liang, Ming Yu, Zhengpeng Gong

**Affiliations:** ^1^Department of Clinical Medicine, Guizhou Medical University, Guiyang, China; ^2^Laryngology and Otology, the Affiliated Baiyun Hospital of Guizhou Medical University, Guiyang, China; ^3^Department of Otorhinolaryngology Head and Neck Surgery, Affiliated Hospital of Guizhou Medical University, Guiyang, China

**Keywords:** COVID-19, otorhinolaryngology, Otolaryngological manifestations, SARS-CoV-2, bibliometric analysis, CiteSpace, VOSviewer

## Abstract

**Background:**

Since it began in December 2019, the coronavirus disease 2019 (COVID-19) outbreak has not been completely contained. COVID-19 has attracted the interest of nations throughout the globe. The global coronavirus outbreak has had an especially devastating effect on otolaryngology. The virus is commonly associated with otorhinolaryngological symptoms. COVID-19 research is becoming more common in otorhinolaryngology. Although various studies on covid-19-related Otorhinolaryngology manifestations have been published, there has been no bibliometric analysis of these articles concentrating on COVID-19-related Otorhinolaryngology research.

**Methods:**

Original publications on Otolaryngological symptoms on COVID-19 were extracted from the Social Sciences Citation Index (SSCI) and the Science Citation Index-Expanded (SCI-E) databases in Clarivate Analytics' Web of Science Core Collection (WoSCC) between January 2020 and May 2022. CiteSpace and VOSviewer were utilized to detect and assess the research focus and trends in this field by extracting the country/region, institution, author, journal, references, and keywords related to this topic.

**Results:**

A total of 631 journals from 97 countries were included in the total of 1,528 articles. Most of the articles on this topic were published in the United States, which had the most citations and the highest H-index. Huazhong University of Science and Technology is the institution with the largest number of articles in the research of COVID-19-related Otorhinolaryngology diseases. Claire Hopkins was the most prolific author belonging to Guy's and St. Thomas' NHS Foundation Trust. Huang CL from Jin Yin-tan Hospital received the most citations among all authors. The most cited article was *Clinical features of patients infected with 2019 novel coronavirus in Wuhan, China*, which was created by Huang CL. Most of the studies relating to COVID-19 and Otorhinolaryngology diseases were published in the European Archives of Oto-Rhino-Laryngology.

**Conclusion:**

COVID-related research in the field of otorhinolaryngology has been studied in terms of descriptive quantitative metrics, which show that academics from around the world are working together to combat this pandemic.

## Introduction

The rapid international spread of SARS-CoV-2, a coronavirus that produces COVID-19, has triggered the COVID-19 pandemic. COVID-19 is a new strain of coronavirus that has never been discovered in people before; it is the seventh known coronavirus that may infect humans. It was identified by the WHO on January 12, 2020 after being identified in a Wuhan viral pneumonia case in 2019 (1, 2). The infection has been found in more than 100,000 people, and it can affect people of any age.

COVID-19 has been confirmed to have human-to-human transmission characteristics and a high level of concealment (1, 3). It can also spread through droplets, direct contact, and even aerosols (4). The coronavirus is characterized mostly by symptoms associated with the lower respiratory tract, such as fever, cough, dyspnea, and chest tightness, which could swiftly escalate to acute respiratory distress syndrome (ARDS) (5). COVID-19 produces a variety of upper respiratory tract symptoms, including nasal congestion, sore throat, and impaired smell. Given the high COVID-19 virus titers in the nasopharynx and other surrounding mucosal surfaces, otolaryngology is viewed as a particularly high-risk field for exposure. A scientometric analysis of COVID19 research relevant to otolaryngology is warranted in light of the expanding international interest in the field. Quantitative data analysis like this will help guide future otolaryngology studies. So far, no bibliometric study has been conducted to examine the growth of COVID19-related otolaryngology research.

Bibliometric analysis is a useful navigational tool for a vast field of scientific data. Its major objective is to conduct a quantitative and qualitative study of publications in a specific research field. With the aid of bibliometric analysis, it is feasible to describe the most important publications and general publishing patterns within a certain scientific field (6). In a comprehensive assessment of academic literature, bibliometric analysis has been widely employed to identify the hot topics and contributions of scholars, journals, and countries/regions (7). The academic infrastructure and research trends of COVID-19-related Otolaryngological conditions can be better understood with the use of a thorough bibliometric analysis. In light of these considerations, we present a systematic bibliometric analysis of the literature.

## Materials and methods

### Search strategy and inclusion criteria

**Figure 1:**
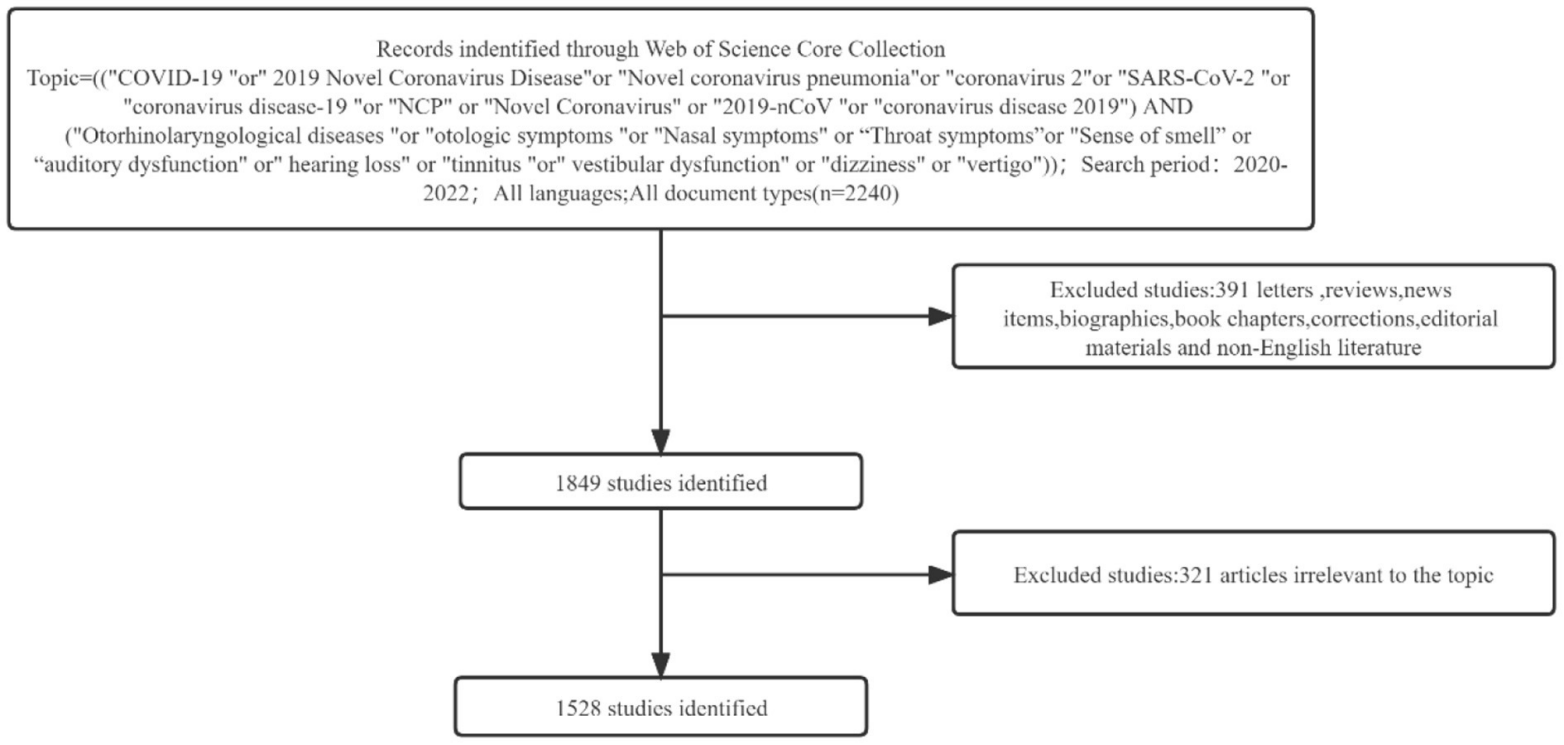
Search strategy framework flowchart.

For our search, we chose the Web of Science database, which is often used for bibliometric analysis due to its comprehensive assessment of publications and high-quality literature (8). The reviewers (JW, SL, MY) independently assessed the article titles, abstracts, and, if necessary, the whole texts for inclusion. Disagreements were handled by conversations among them and, if required, with a fourth reviewer (ZPG) in the event that an agreement could not be reached. The reviewers extracted material separately from the included papers. Discrepancies were subsequently managed through reviewer discussion. To avoid mistakes caused by database upgrades, on May 16, 2022, all data were retrieved and exported. The search terms were TS = (COVID-19 or 2019 Novel Coronavirus Disease or Novel coronavirus pneumonia or coronavirus 2 or SARS-CoV-2 or coronavirus disease-19 or NCP or Novel Coronavirus or 2019-nCoV or coronavirus disease 2019) AND TS = (Otorhinolaryngological diseases or otologic symptoms or Nasal symptoms or Throat symptoms or Sense of smell or auditory dysfunction or hearing loss or tinnitus or vestibular dysfunction or dizziness or vertigo). We conducted a comprehensive search using only these terms to guarantee that relevant literature was included. The duration of this span is from January 2020 to May 2022. English-language publications with original content met the inclusion criteria. Aside from publications that did not contain unique content, articles written in languages other than English were ruled out. The search and download operation was conducted on May 16, 2022, in an effort to eliminate large errors caused by routine database changes. Figure 1 provides detailed information on enrolment and selection. Since the data is retrieved straight from the database, there is no need for ethical approval.

### Bibliometric analysis

The data from these articles were imported and combined using an online analysis platform for literature metrology (http://bibliometric.com/). Two tools were used to depict the network of this data: Drexel's CiteSpace v5.8.R3 SE, 64-bit (Philadelphia, PA, United States) and Leiden's VOSviewer v1.6.18 (Leiden University, Leiden, the Netherlands). They were utilized to show the network of this data, including counties, authors, institutions, and journals (9, 10). The software VOSviewer was used to investigate the correlations between the most productive nations, research institutions, and commonly used terms. CiteSpace, a publicly accessible Java program, was created to study and display trends and patterns in scientific publications, demonstrating the structure and distribution of scientific knowledge. Besides it was used to do cluster analysis and generate social network maps (consisting of nodes and linkages) for nations, institutions, and keywords. Titles, authors, co-cited authors, journal sources, keywords, author affiliations, and the nations or regions to which the authors belong were tabulated as publication characteristics. Co-cited writers denote authors who have been cited together. Two significant author level metrics are the Hirsch index (H-index) and the total number of citations (total times cited count). According to the H-Index, at least h out of N journal articles have been referenced at least h times in the literature (11). It is a statistic that attempts to quantify a scientist's papers' productivity and citation impact.

## Results

### Data analysis

**Figure 2:**
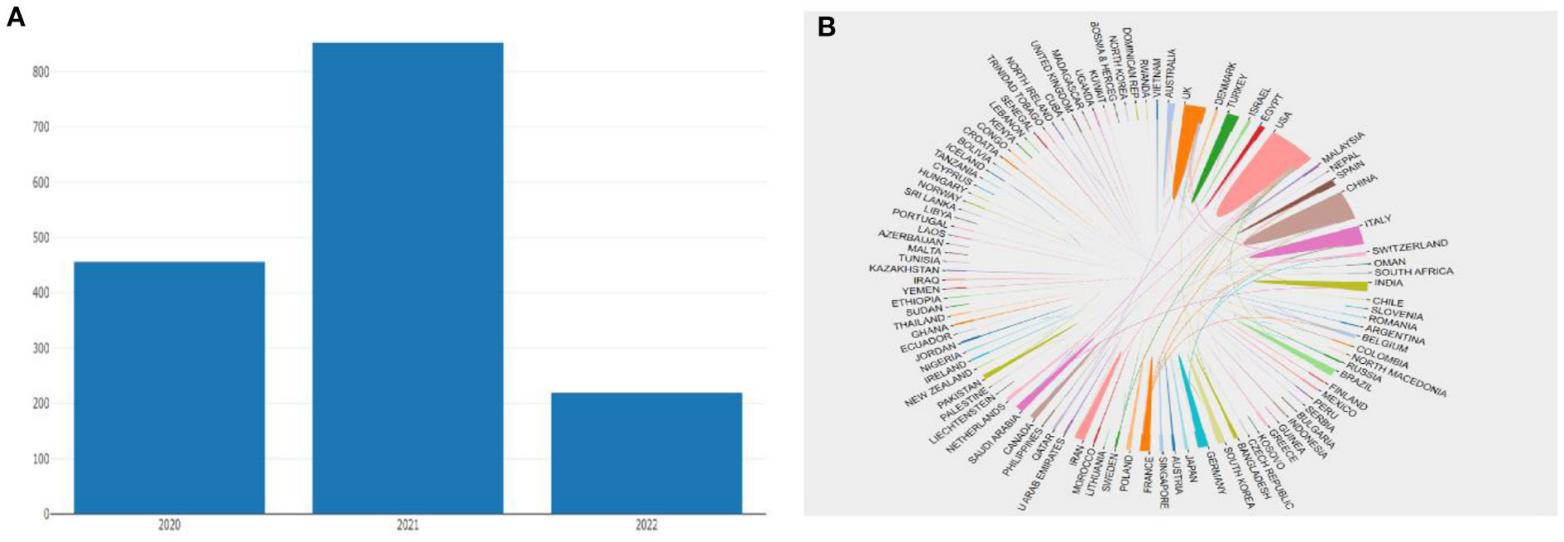
**(A)** Annual number of publications. **(B)** The geographic dispersion of nations/regions. Cooperation between nations/regions.

The Core Collection Database of Web of Science was queried for a total of 2,240 papers on COVID-19 and Otorhinolaryngology-related disorders. Non-English studies, early access, new items, reviews, and books were omitted. Figure 2A shows the annual volume of publications for the relevant studies. Ultimately, 1,528 articles were accounted for 631 publications from 97 countries contributed to these works. The number of research articles in pertinent areas was 456 in 2020, 852 in 2021, 220 as of January 1, 2022, and May 16, 2022, as of the search date. The network of national cooperation for COVID-19-related Otorhinolaryngology research is depicted in Figure 2B. The low density of the national research network map indicates that the research team is relatively independent and underlines the need for more collaboration (Table 1).

**Table 1:** Top 10 countries publishing research articles.

**Country**	**Publications**	**Total citations**	**Total link strength**	**Centrality**	**H-index**
USA	348	12,123	1,863	0.14	38
China	210	27,030	1,196	0.01	34
Italy	147	4,569	1,335	0.03	22
England	146	5,311	1,247	0.14	28
Germany	91	3,145	636	0.05	16
Turkey	87	642	498	0	11
India	77	2,322	547	0.09	13
France	64	3,020	869	0.12	17
Brazil	58	833	275	0.01	11
Iran	57	1,068	379	0.05	9

### Countries with the top 10 articles

**Figure 3:**
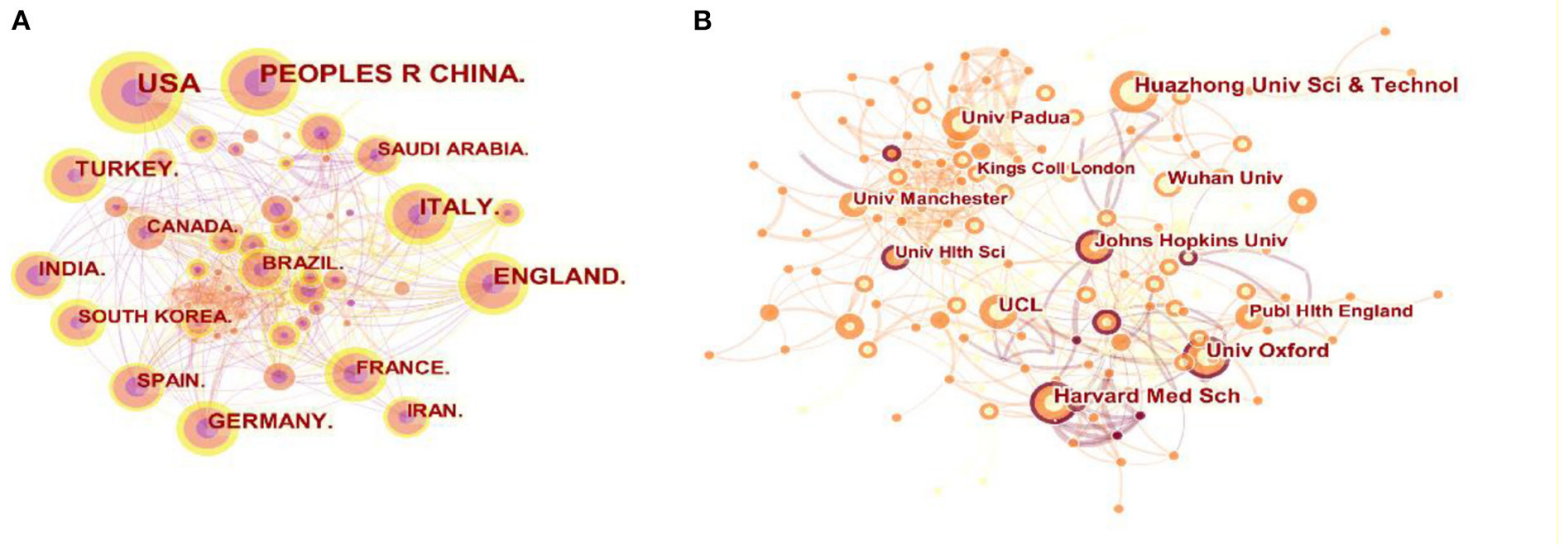
Visualization map of the scientific collaboration network analysis of COVID-19 and otorhinolaryngology research field. Collaboration among countries/regions **(A)** and institutions **(B)**.

Table 1 shows the most often cited countries that publish in the journal, as well as the relationships between countries in terms of how often they are cited. The table shows that the countries with the most publications are the United States (22.7%), China (13.7%), and Italy (9.6%). The centrality index is a measure of the relevance of network nodes in a network. Higher centrality in a collaborative network correlated to more intensive collaboration. According to centrality analysis, the United States (0.14) and the United Kingdom (0.14) were at the center of the network, followed by France (0.12). Figure 3A illustrates the top 10 contributing countries in this field. The size of the circle indicates the number of articles, with the circle growing larger as the number of posts increases, and the lines between the elements represent the cooperative relationship between countries.

### The top 10 institutions with the most articles

Among the 10 institutions with the most articles, four were in the United States, three were in the United Kingdom, two were in China, and one was in Italy (Figure 3B). The map's nodes represent elements institute, and the connecting lines between nodes represent collaboration ties. The wider the circle, the more articles that have been published. The wider the line, the stronger the bond. Of these institutions, Huazhong University of Science and Technology (32 articles) in China contributed the most articles with a centrality of 0.11, followed by Harvard Medical School in the United States (28 articles), with the highest centrality (0.22) (Table 2).

**Table 2:** Top 10 institutions publishing research articles.

**Rank**	**Institution**	**Publications**	**Centrality**	**Country**
1	Huazhong University of Science and Technology	32	0.11	China
2	Harvard Medical School	28	0.22	United States
3	University of Oxford	23	0.07	United Kingdom
4	University College London	23	0.11	United Kingdom
5	Wuhan University	22	0.05	China
6	Johns Hopkins University	21	0.14	United States
7	University of Padua	20	0.01	Italy
8	Public Health England	17	0	United Kingdom
9	University of Manchester	16	0.15	United States
10	University of Texas Health Science Center in Houston	14	0.01	United States

### The top 10 authors with the largest number of publications and most cited authors

**Figure 4:**
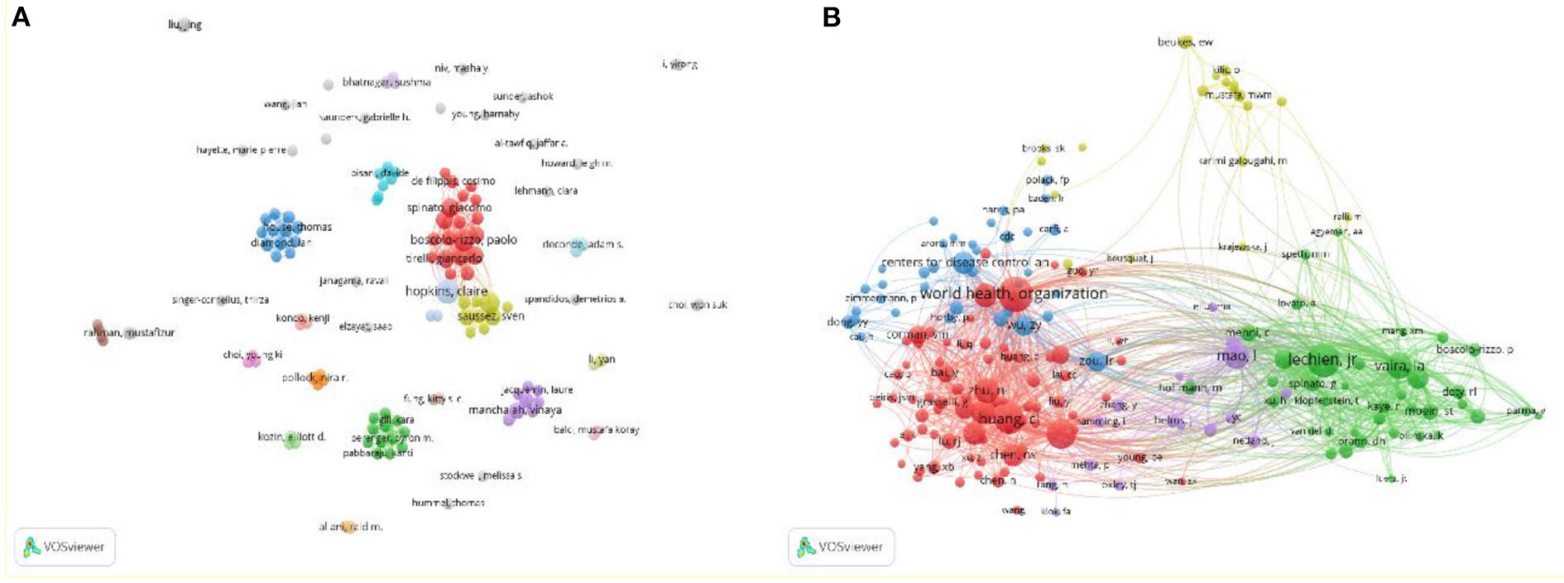
Visualization map of the scientific collaboration network analysis of COVID-19-related otorhinolaryngology research field. Collaboration among author **(A)** and co-cited author **(B)**.

The details of the top 10 authors with the most published articles and the top 10 authors with the most citations are shown in Table 3. They published 71 papers and accounted for 4.6% of the total papers. Claire Hopkins from ENT Department, Guy's Hospital, London was the most productive author in this scope with 15 publications and a high centrality (0.01). Followed by Jerome R Lechien from the Paris Saclay University with 9 papers. In terms of co-cited authors, Huang CL, Guan W, and Lechien JR were ranked the top three. Among them, Huang CL has the highest centrality (0.08). The supplementary figure showed the author cooperation network (Figure 4A) and co-cited author network (Figure 4B). There were scattered co-operations between them, and authors who work together have strong citation ties.

**Table 3:** Top 10 authors and co-cited authors involved in research.

**Rank**	**Author**	**Country**	**Publications**	**Centrality**	**Co-cited Author**	**Country**	**Co-citations**	**Centrality**
1	CLAIRE HOPKINS	United Kingdom	15	0.01	Huang CL	China	273	0.08
2	Jerome R. Lechien	France	9	0	Guan W	China	262	0.05
3	Sven Saussez	Belgium	7	0	Lechien JR	Belgium	246	0.1
4	Paolo Boscolorizzo	Italy	7	0	Mao L	China	234	0.22
5	Carlos M. Chiesa-Estomba	Spain	6	0	Zhu N	China	223	0
6	Cristoforo Fabbris	Italy	6	0	Wang DW	China	175	0.02
7	Cosimo De Filippis	Italy	6	0.01	World Health Organization	N/A	161	0
8	Giacomo Spinato	Italy	5	0	Chen NS	United Kingdom	159	0.01
9	Anna Menegaldo	Italy	5	0	Zhou F	China	142	0.01
10	Vinaya Manchaiah	United States	5	0	Chan JFW	China	139	0.01

### The top 10 frequently cited articles

**Figure 5:**
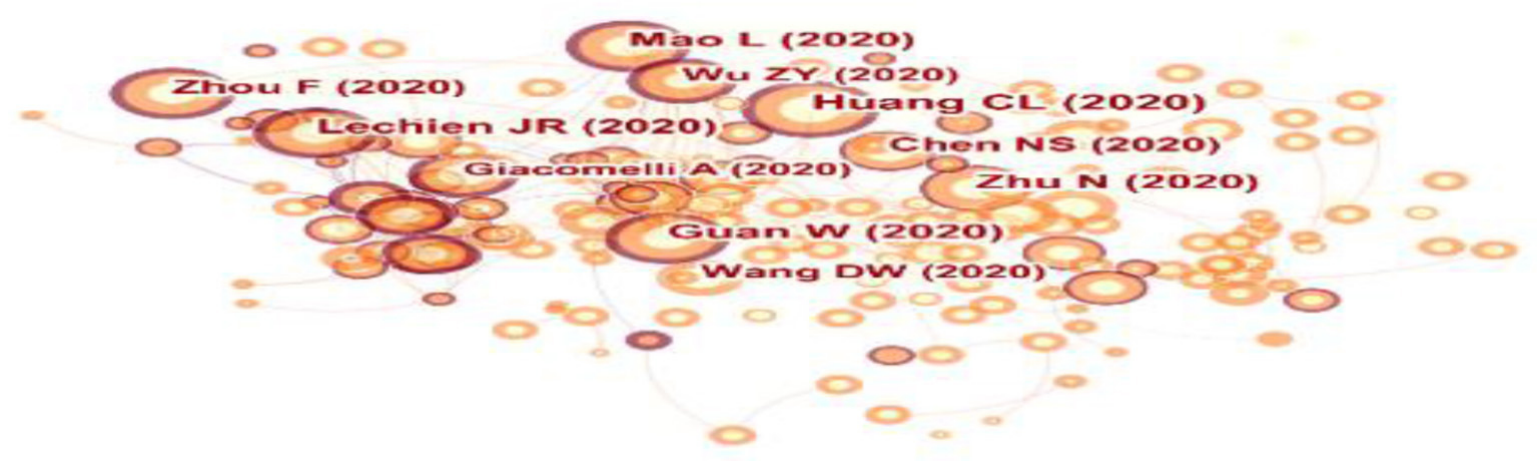
An interconnected web of citations.

Citations are frequently regarded as an essential component of the bibliometric study. Figure 5 illustrates the document co-citation network using CiteSpace software. Nodes indicate citations in data sources, while linkages reflect co-citations between publications. As two references are referenced together, the line that connects them shows how frequently they are referred to by other sources. The article with the greatest number of citations on this topic worldwide was *Clinical features of patients infected with 2019 novel coronavirus in Wuhan, China*, which was submitted by Huang CL in China, and this article was cited 248 times in this research. Eight of the top ten most referenced articles on this topic originated in China. It should be noted that three of the top ten most referenced pieces were published in The Lancet's special COVID-19 sections, two in The New England Journal of Medicine, and five of them had an IF of more than 50 (Table 4).

**Table 4:** Top 10 most frequently cited articles.

**Authors**	**Article title**	**Citation count**	**Publication date**	**Quartile in category**	**Impact factor (IF) (2020)**	**Journal**
Huang et al. (12)	Clinical features of patients infected with 2019 novel coronavirus in Wuhan, China	248	February 15, 2020	Q1	79.323	Lancet
Guan et al. (13)	Clinical Characteristics of Coronavirus Disease 2019 in China	221	April 30, 2020	Q1	91.253	New Engl J Med
Mao et al. (14)	Neurologic Manifestations of Hospitalized Patients With Coronavirus Disease 2019 in Wuhan, China	197	June 1, 2020	Q1	18.302	Jama Neurol
Lechien et al. (15)	Olfactory and gustatory dysfunctions as a clinical presentation of mild-to-moderate forms of the coronavirus disease (COVID-19): a multicenter European study	183	April 6, 2020	Q2	2.503	European Archives of Oto-Rhino-Laryngology
Zhu et al. (1)	A Novel Coronavirus from Patients with Pneumonia in China, 2019	169	February 20, 2020	Q1	91.253	New Engl J Med
Chen et al. (16)	Epidemiological and clinical characteristics of 99 cases of 2019 novel coronavirus pneumonia in Wuhan, China: a descriptive study	142	February 15, 2020	Q1	79.323	Lancet
Zhou et al. (17)	Clinical course and risk factors for mortality of adult inpatients with COVID-19 in Wuhan, China: a retrospective cohort study	139	March 28, 2020	Q1	79.323	Lancet
Wang et al. (18)	Clinical Characteristics of 138 Hospitalized Patients With 2019 Novel Coronavirus-Infected Pneumonia in Wuhan, China.	120	March 17, 2020	Q2	2.743	Jama-J Am Med Assoc
Wu and McGoogan (19)	Characteristics of and Important Lessons From the Coronavirus Disease 2019 (COVID-19) Outbreak in China: Summary of a Report of 72 314 Cases From the Chinese Center for Disease Control and Prevention.	117	April 7, 2020	Q2	2.743	Jama-J Am Med Assoc
Giacomelli et al. (20)	Self-reported Olfactory and Taste Disorders in Patients With Severe Acute Respiratory Coronavirus 2 Infection: A Cross-sectional Study.	109	July 28, 2020	Q1	9.079	Clin Infect Dis

### Ten journals with the largest number of articles and the journals with the most citations

**Figure 6:**
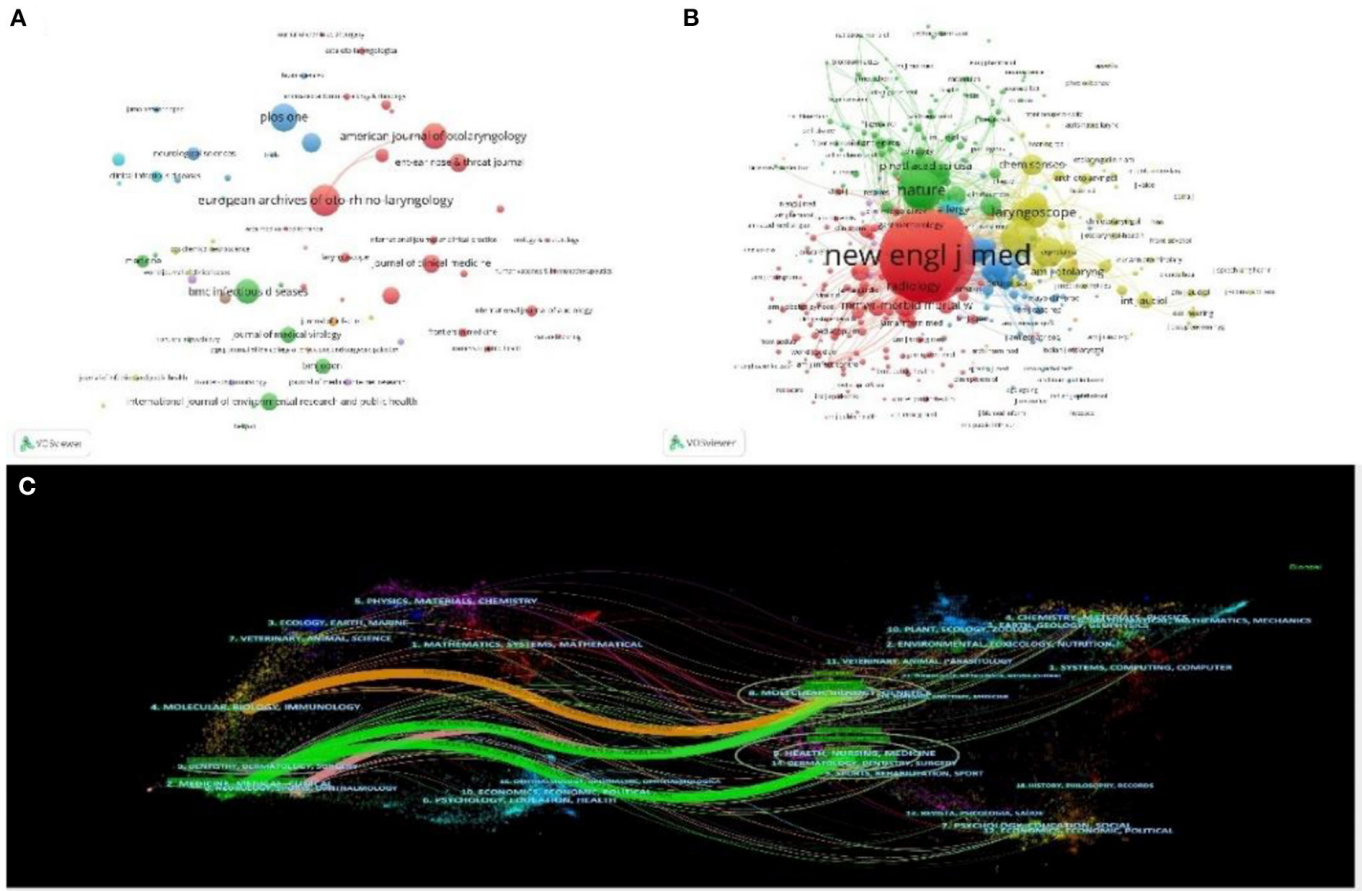
Visual map analysis of the network relationship map of published **(A)** and cited magazines **(B)** in COVID-19-related otorhinolaryngology research. **(C)** The dual-map overlay of journals contributed to publications on COVID-19-related otorhinolaryngology research.

The 10 journals with the greatest number of published articles are shown in Table 5, with details including the number of articles, impact factor, total citations and country of origin. European Archives of Oto-Rhino-Laryngology was the top journal with 34 published articles. The journal's impact factor is 2.503, and there were 321 citations. Plos One was the second most active journal, followed by American Journal of Otolaryngology, BMC Infectious Diseases, and Frontiers in Neurology. Journal of Clinical Medicine has the highest impact factor with 4.242. The top three cited journals are with The New England Journal of Medicine, Lancet, and Jama-Journal of The American Medical Association, with 1,963, 1,668, and 1,264 co-citations, respectively (Table 5). The relationship network diagram of the published journals (Figure 6A) and the cited journals are shown in Figure 6B. The dual-map overlay of journals is shown in Figure 6C, with the citing journals on the left side, cited journals on the right side, and the colored paths indicating the citation relationships. Journals' disciplines are represented by labels. Colored lines represent the reference path from left to right. There are four different ways to quote. The two green citation paths show that the research of molecular/biology/genetics journals and health/nursing/medicine journals are often cited by medicine/medical/clinical journals. The orange path indicates that research in molecular/biology/genetics journals is often cited by research in molecular/biology/immunology journals. The pink path indicates that research in molecular/biology/genetics journals is often cited by research in neurology/sports/ophthalmology journals.

**Table 5:** Top 10 journals by several publications and co-citations.

**Rank**	**Journal**	**Publications**	**Country**	**IF (2020)**	**Cited journal**	**Citations**	**Country**	**IF (2020)**
1	European Archives of Oto-Rhino-Laryngology	34	GERMANY	2.503	The New England Journal of Medicine	1,963	United States	91.253
2	Plos One	30	USA	3.24	Lancet	1,668	United Kingdom	79.323
3	American Journal of Otolaryngology	28	USA	1.808	Jama-Journal of The American Medical Association	1,264	United States	56.274
4	BMC Infectious Diseases	27	ENGLAND	3.09	Journal of Medical Virology	826	United States	2.327
5	Frontiers in Neurology	21	SWITZERLAND	4.003	Nature	599	United Kingdom	49.962
6	Journal of Clinical Medicine	20	SWITZERLAND	4.242	Plos One	534	United States	3.24
7	Ent-Ear Nose & Throat Journal	20	USA	1.697	European Archives of Oto-Rhino-Laryngology	522	GERMANY	2.503
8	International Journal of Infectious Diseases	19	ENGLAND	3.623	Lancet Infectious Diseases	511	United States	25.071
9	International Journal of Environmental Research And Public Health	19	SWITZERLAND	3.39	Journal of Virology	457	United States	5.103
10	Journal of Medical Virology	18	USA	2.327	International Journal of Infectious Diseases	445	United Kingdom	3.623

### The top 10 areas of research

**Figure 7:**
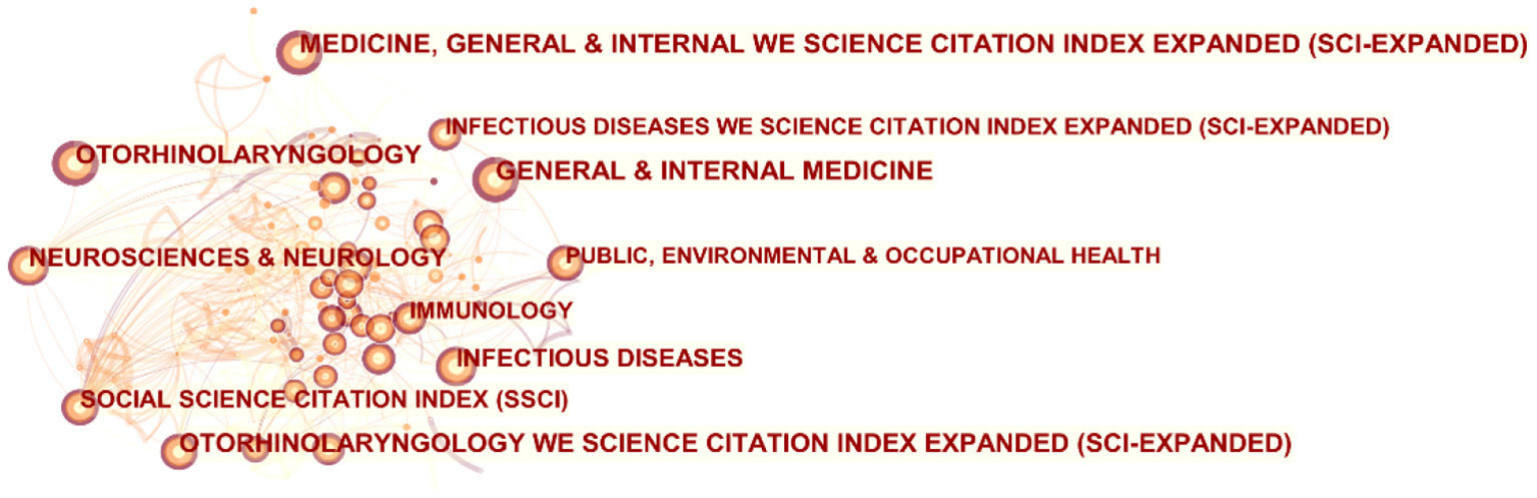
Visual analysis of network diagram in COVID-19-related otorhinolaryngology areas of research.

The area of research is multidisciplinary. However, the top five fields are general & internal medicine, medicine general and internal we science citation index expanded (sci-expanded), otorhinolaryngology, otorhinolaryngology we science citation index expanded (sci-expanded), infectious diseases (Table 6) are particularly worth mentioning. It is worth pointing out that the Social Science Citation Index (SSCI) is with the highest centrality (0.64). More supplementary information is shown in Figure 7.

**Table 6:** The 10 research areas with the highest number of publications.

**Category**	**Frequency**	**Centrality**	**Degree**	**Half-Life**
General and Internal Medicine	244	0.05	21	0.5
Medicine, General and Internal We Science Citation Index Expanded (Sci-Expanded)	216	0	4	0.5
Otorhinolaryngology	192	0.02	13	0.5
Otorhinolaryngology We Science Citation Index Expanded (Sci-Expanded)	169	0	8	0.5
Infectious Diseases	163	0.06	22	0.5
Neurosciences and Neurology	151	0.14	36	0.5
Social Science Citation Index (SSCI)	132	0.64	73	0.5
Immunology	99	0.1	28	0.5
Infectious Diseases We Science Citation Index Expanded (Sci-Expanded)	95	0	3	0.5
Public, Environmental and Occupational Health	89	0.05	21	0.5

### Keywords analysis

**Figure 8:**
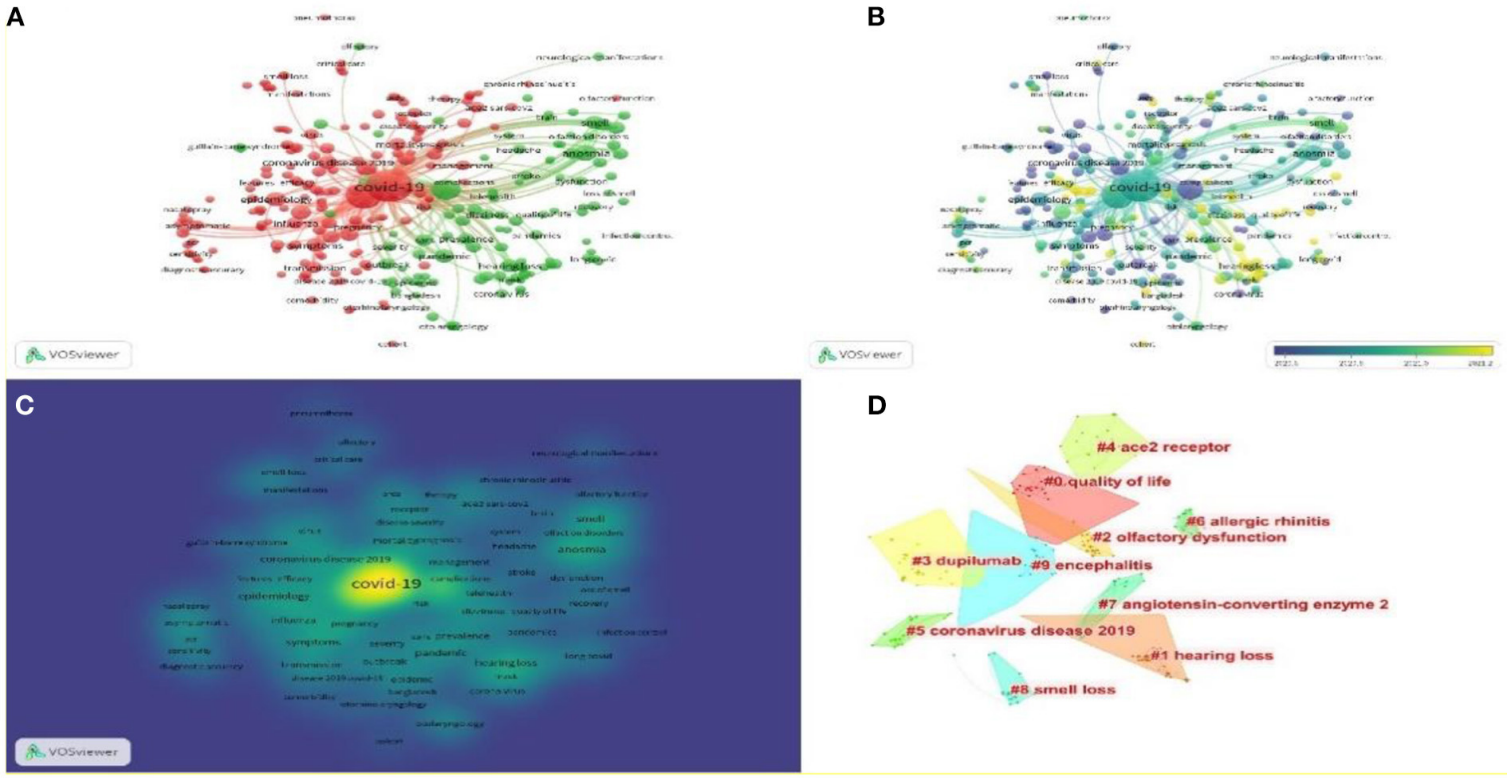
Bibliometric analysis of keywords for COVID-19 research in otorhinolaryngology. **(A)** Keywords distribution. **(B)** Map showing trending themes from June 2020 to February 2021 based on keywords. From purple to yellow, the indicator reflects the current status of publications. To determine how frequently two objects appear together, the distance between two circles is plotted against the frequency of appearance. **(C)** The density of keywords on a map. **(D)** Analysis of keyword clusters (the order is from #0 to #9; The smaller the number, the more keywords the cluster contains, so the more attention it receives).

The keywords selected by the author when submitting the manuscript for publication are extracted by the VOSviewer. As shown in Figure 8A, 136 keywords appeared more than 10 times in the titles and abstracts of all articles during the analysis. The most used keywords were “COVID-19” (1,058 occurrences), “SARS-CoV-2” (456 occurrences), “Coronavirus” (226 occurrences), and “anosmia” (100 occurrences). Table 7 and Figure 8 display the most used keywords in the relevant publications, and they are coupled with other keywords that appear in the publication and their frequency. As shown in Figure 8B, the VOSviewer colors all keywords based on the average number of occurrences of each word. In particular, purple indicates that the word appears relatively early, while yellow indicates a relatively new appearance, which to some extent reflects the change of focus and future research trends. Figure 8C shows the hot topics of the research. Keyword clustering is analyzed by CiteSpace, and Figure 8D shows the top 10 keyword clusters.

**Table 7:** Top 20 most used keywords.

**Keyword**	**Frequency**	**Centrality**	**Total link strength**
Covid-19	1,058	0.07	3,095
SARS-CoV-2	456	0.08	1,577
Coronavirus	226	0.02	932
Anosmia	100	0.03	486
Infection	90	0.09	397
Pneumonia	73	0.04	298
Children	66	0.05	273
Smell	63	0.03	228
Symptoms	59	0.08	253
Pandemic	58	0.03	283
Epidemiology	51	0.09	195
Clinical characteristics	51	0.01	188
Taste	50	0.01	209
Wuhan	47	0.01	219
Coronavirus disease 2019	42	0.07	142
Mortality	38	0.06	166
Hearing loss	37	0.06	212
Olfactory dysfunction	37	0.03	180
Outbreak	32	0.03	141
Tinnitus	30	0.01	136

## Discussion

The main purpose of this study is to use CiteSpace and VOSviewer for network analysis and visualization to analyze COVID-19 research in otorhinolaryngology in the existing WOS literature. Even though COVID-19 continues to spread over the world, the fight against the disease continues. There's a pandemic going on right now. Researchers from all over the world must pay close attention to the symptoms of COVID-19 because of the virus's ongoing reappearance and evolution. Sneezing, coughing, or conversing with someone who is infected is thought to spread the virus (21). COVID-19 virus enters and reproduces in the nasal cavities of humans. COVID-19's symptoms in otorhinolaryngology are significant in early detection because the nasal cavity is the virus's entrance point. At present, there is no bibliometric study on the manifestation of COVID-19-related symptoms in otorhinolaryngology diseases.

In this study, the United States published the most articles between January 1, 2020, and May 16, 2022, followed by China, Italy, and the United Kingdom (Table 1). Among these countries, China was the first nation to identify and report the COVID-19 virus. And the United States has had the newest coronavirus infections and deaths (22). In terms of international collaboration, the United States cooperates with most nations and with the highest H-index (23). In terms of institutions, the top three institutions were Huazhong University of Science and Technology, the University of Oxford, and Harvard Medical School. As the city where the COVID-19 epidemic first occurred, first-hand medical cases and treatment experiences are available in Wuhan (24), so Huazhong University of Science and Technology in Wuhan has published a relatively large number of articles. Although COVID-19 is popular all over the world, except for China, the top 10 publishers are all from the United States, the United Kingdom, and Italy, which is related to the advanced level of medical research in Europe and North America. In addition to identifying top researchers, this study can also evaluate and illustrate research partnerships among researchers. Claire Hopkins has produced the most publications in the COVID-19-related otorhinolaryngology research. Furthermore, Jerome R Lechien, Sven Saussez, Paolo Boscolorizzo, and Carlos M Chiesa-Estomba were the top five most productive authors. Claire Hopkins and Cosimo De Filippis were the top two authors with a high centrality (0.01). However, it is crucial to note that scholars focusing on COVID-19-related worldwide otorhinolaryngology research have a distinct regional profile, with the majority of these academics based in Europe and the US. These authors primarily work in the otolaryngology departments of the hospital connected to their universities. Therefore, improving communication and collaboration among international academics would aid in the development of COVID-19 research connected to otorhinolaryngology research.

The most published journals in COVID-19-related otorhinolaryngology research are the leading journals in the field of general and internal medicine and otolaryngology, including European Archives of Oto-Rhino-Laryngology, Plos One, American Journal of Otolaryngology, BMC Infectious Diseases, and Frontiers in Neurology. These journals have had a significant influence on otorhinolaryngologists, infection physicians, and neurologist across the world, and have impacted the orientation of research in their corresponding scientific fields. This trend indicates that COVID-19-related Otorhinolaryngology is one of the central issues in otolaryngology, physician, and neurologist. The top 10 co-cited references for the period 2020-2022 indicated that researchers are more concerned with the clinical management of COVID-19-related Otorhinolaryngology research. Notably, the first reference with the highest co-citation rate and landmark was the article “Clinical features of patients infected with 2019 novel coronavirus in Wuhan, China” published by Huang C (12), which proposed the majority of patients reported fever, dry cough, dyspnea, and bilateral ground-glass opacities. These characteristics of 2019-nCoV infection resemble those of SARS-CoV and MERS-CoV infections. Besides, some individuals infected with 2019-nCoV showed notable upper respiratory tract symptoms, such as rhinorrhea, sneezing, and sore throat.

The analysis of keyword co-occurrence provides insight into the distribution and evolution of various research hotspots within a given topic. The purpose of keyword co-occurrence from a micro-perspective is to address particular questions in the subject area. As a highly simplified form of the paper's content, keywords can, to some extent, immediately and simply convey the paper's theme. The keyword co-occurrence network is an analytical method based on text content. Examining the co-occurrence of keyword pairs within the same text sorts out the relationships between the many themes of the discipline and helps readers become more familiar with it. The figure depicts the results of the co-occurrence of terms in Otorhinolaryngology papers associated with COVID-19. We found that “covid-19,” “SARS-CoV-2,” “coronavirus,” “anosmia,” and “infection” are the five most frequent keywords.

Figure 8D shows the top 10 keywords clustering based on the log-likelihood rate (LLR) algorithm. They encompass plenty of concerns in the field of COVID-19-related Otorhinolaryngology research, comprising “#0 quality of life,” “#1 hearing loss,” “#2 olfactory dysfunction,” “#3 dupilumab,” “#4 ace2 receptor,” “#5 coronavirus disease 2019,” “#6 allergic rhinitis,” “#7 angiotensin-converting enzyme 2,” “#8 smell loss,” “#9 encephalitis.” COVID-19-related quality of life, pathophysiological mechanisms, and Otorhinolaryngology-related symptoms are still hotspots in the study of COVID-19 ENT-related symptoms.

COVID-19 which is the name of the sickness caused by the SARS-CoV-2 virus persists to disturb our life as a pandemic in 2020 (1). It is well-known that the condition can be asymptomatic or cause mild to severe symptoms. According to data from recent studies, the severity of the disease varies epidemiologically by race, gender, and age (19). COVID-19 patients could have symptoms like coughing, shortness of breath, sore throat, rhinorrhea, nasal congestion, throat congestion, swollen tonsils, enlarged lymph nodes in the neck, or dizziness (12). COVID-19 affects people of all ages, and most organ systems are vulnerable to infections. The keyword cluster “#0 quality of life” in this study indicates that as COVID-19 has continued to spread, researchers have increasingly become interested in the influence of the virus on people's quality of life. Previous research has indicated that COVID-19 infection has a significant impact on stress, sadness, and anxiety (25). Due to significant uncertainty and worry, the COVID-19 pandemic has worsened psychological wellness (26). In addition, persons encountered huge changes in their everyday lives, such as social isolation and home confinement, which dramatically reduced health-related quality of life (HRQoL) (27). In this context, earlier research has demonstrated that HRQoL has decreased as a result of the COVID-19 pandemic. There has been increased attention on the psychological impact of disease both during and after discharge from the hospital in more recent publications (27–29). Post-acute COVID-19 patients in rehabilitation were still experiencing physical and psychological symptoms, and their HRQoL was significantly lower (27, 30). Patients with COVID-19 may have fatigue, anxiety, sadness, post-traumatic stress disorder (PTSD), and cognitive dysfunction in the long run, according to follow-up studies (31–34). To give effective care to patients throughout the acute phase of the disease and also during follow-up after COVID-19 infection, health care practitioners must be aware of these differences in the disease course (27, 32).

COVID-19 ENT-related symptoms such as anosmia, smell, taste, hearing loss, taste disorder, olfactory dysfunction, and tinnitus appeared in the keywords occurrences of this study. In keyword clusters, we can see cluster words such as “hearing loss,” “olfactory dysfunction,” “allergic rhinitis,” and “smell loss.” Evidence suggests that the novel coronavirus, which is thought to be the origin of the COVID-19 epidemic, also reduces patients' senses of smell and taste (35). Hyposmia/anosmia was reported in a considerable proportion of COVID-19 patients. Additionally, reports are indicating that COVID-19 may manifest as isolated anosmia (36). These patients may be the source of COVID-19's rapid dissemination. The deterioration of olfactory function in asymptomatic patients has generated interest as a possible early indicator of SARS-CoV-2 infection (37). The development of abrupt loss of smell and taste was rapidly identified as one of COVID-19's symptoms (18). In the past, olfactory impairment has been recorded following SARS-CoV infection. This single-stranded RNA virus uses the enzyme angiotensin-converting enzyme 2 (ACE2) as a receptor. ACE2, which is found throughout the human respiratory system, is responsible for SARS-capacity CoV-2's to infect humans through the respiratory tract (23, 38, 39). Nasal epithelial cells, specifically goblet and ciliated cells, have been implicated in molecular studies as the entry point for severe acute respiratory syndrome coronavirus 2 (SARS-CoV-2) and a reservoir for transmission within a given patient and from person to person. According to research, allergic rhinitis may be a protective factor against COVID-19 infection (40). This could be because allergen stimulation of the respiratory tract can cause allergic airway inflammation, which leads to decreased production of angiotensin converting enzyme 2, implying that allergic inflammation may have a substantial role in lowering the risk of COVID-19 infection (41). Moreover, due to the high viral load in the nasal cavity, olfactory neurons may be especially sensitive to injury (17). Olfactory sensory neurons (OSN), whose cilia emerge from the nasal cavity and whose axons develop into the olfactory bulb, connect the respiratory tract directly to the central nervous system (CNS) (42). COVID-19 has a characteristic loss of smell and various respiratory viruses (influenza, endemic human CoVs, SARS-CoV-1) infiltrate the CNS via a retrograde route, therefore some research speculate postulated that SARS-CoV-2 would be neurotropic and capable of invading the CNS through OSNs (36, 43). The presence of ACE2 receptors in the brain, medulla oblongata, and temporal lobe promoted the entry of SARS-CoV-2 into the brainstem and hearing centers, resulting in the production of inflammatory cytokines (44). Infection with the SARS-CoV-2 virus can result in a wide spectrum of extrapulmonary, sensory, and brain consequences, such as abrupt development of olfactory and gustatory dysfunction, otologic symptoms, and long-term neurological complications (45–48). Hearing impairment may be caused by viruses, immunological complexes, vascular blockage, or cellular stress response (49). It is believed that the SARS-CoV-2 virus causes sensorineural hearing loss by an inflammatory response on cochlear hair cells (47, 50).

## Conclusions

COVID-19's otorhinolaryngology-related disorders, including their clinical manifestations and possible molecular mechanisms, are the primary goals of this work. Early symptoms of COVID-19 may include hearing loss, tinnitus or dizziness/vertigo, and smell or taste disorders; these individuals may be ignored, allowing the virus to propagate unnoticed. Paying attention to otorhinolaryngology symptoms can assist in finding infected people early, classifying patients based on disease severity, and developing a prevention and treatment system. Besides, otolaryngologists are at a high risk of becoming infected and should thus take the necessary precautions in dealing with patients. Moreover, the quality of life associated with COVID-19 has steadily garnered the interest of researchers. Our bibliometric analysis gives descriptive and quantitative metrics for COVID-related research in the field of otorhinolaryngology and shows that researchers around the world are working together more to fight this pandemic.

### Limitations

This study examined papers from the WOS database and sought objective and dependable results. Due to the fact that the search is limited to English research and the database is continually updated, as well as the exclusion of non-research articles, the results may differ somewhat from the actual findings. For more exhaustive results, you can conduct additional research using databases such as Medline, Scopus, or Google Scholar. In addition, variations in databases that are regularly updated may cause discrepancies between search results and the actual number of publications included. Excluding books, chapters, and letters and examining just English-language articles may result in some departures from the findings. The full investigation of diseases from the perspectives of epidemiology and statistics will receive more focus. However, early and prompt detection of COVID-19-related otorhinolaryngology symptoms, the investigation of potential molecular pathways, and the development of effective treatments remain the focus of research. The optimal solution to the COVID-19 epidemic necessitates the collaboration of all disciplines.

## Data availability statement

The original contributions presented in the study are included in the article/supplementary material, further inquiries can be directed to the corresponding author/s.

## Author contributions

The study was created by JW and SL. The data was interpreted and written by JW. The data was collected and examined by SL, MY, and ZG. MY double-checked the data. The article was revised by ZG and MY. The final manuscript was read and approved by all authors.

## Funding

This study was supported by the Science and Technology Foundation of Guizhou Provincial Health Commission (Grant No. 2020XMSB00026375) and the Science and Technology Foundation of Guizhou Provincial (Grant No. The basis of Guizhou Science and Technology Cooperation-ZK[2022]-General 411). The funders had no role in the study's design, the collection, analysis, and interpretation of the data, and the writing of the manuscript.

## Conflict of interest

The authors declare that the research was conducted in the absence of any commercial or financial relationships that could be construed as a potential conflict of interest.

## Publisher's note

All claims expressed in this article are solely those of the authors and do not necessarily represent those of their affiliated organizations, or those of the publisher, the editors and the reviewers. Any product that may be evaluated in this article, or claim that may be made by its manufacturer, is not guaranteed or endorsed by the publisher.
